# Gliotic opaque posterior hyaloid membrane separation: report of two cases

**DOI:** 10.1186/s12886-021-02072-5

**Published:** 2021-08-23

**Authors:** Fumio Hosaka, Wataru Saito, Satoru Kase, Susumu Ishida

**Affiliations:** 1 Iwamizawa Municipal General Hospital, Iwamizawa, Japan; 2 Kaimeido Eye and Dental Clinic, Sapporo, Japan; 3 Department of Ophthalmology, Faculty of Medicine, Graduate School of Medicine, Hokkaido University, Nishi 7, Kita 15, Kita-ku, 060-8638 Sapporo, Japan

**Keywords:** Glial fibrillary acidic protein, Gliosis, Posterior hyaloid membrane, Vitreous opacity

## Abstract

**Background:**

To report two cases with idiopathic unilateral diffuse opacification of the posterior hyaloid membrane (PHM) completely separated from the retina, the mechanism of which is possibly due to glial cell proliferation and migration.

**Case presentation:**

Two Japanese women at age 75 and 84 with no systemic or ocular history developed diffuse opacification in one eye resembling a ground glass sheet almost all over the surface of the PHM, but not within the vitreous gel or fluid. The retinas were funduscopically normal; however, optical coherence tomography demonstrated hyperreflective icicle-like anterior protrusions from the surface of the fovea. The patients received pars plana vitrectomy, resulting in visual improvement. Cell block preparations of the vitreous in one case revealed a cluster of cells immunoreactive for glial fibrillary acidic protein in consistence with gliosis, while denying vitreoretinal lymphoma from lack of atypical cells and vitreous amyloidosis due to no staining for Congo red or direct fast scarlet. The lesions did not recur during follow-up with no new funduscopic abnormalities.

**Conclusions:**

To our knowledge, this is the first to demonstrate such peculiar cases of vitreous opacity with idiopathic and unilateral onset. Histological assessments revealed the possible pathogenesis of gliotic opaque PHM separation to cause its ground-glass-sheet appearance.

**Supplementary Information:**

The online version contains supplementary material available at 10.1186/s12886-021-02072-5.

## Background

Vitreous opacity is one of the frequent vision-threatening abnormalities. The causative diseases of vitreous opacity include intraocular inflammation, vitreoretinal lymphoma, and vitreous amyloidosis, each of which develops distinct opaque features resembling snowball in sarcoid uveitis [1], glass wool in vitreous amyloidosis [2], and aurora in vitreoretinal lymphoma [3]. Regardless of the causative diseases, vitreous opacity generally affects the vitreous gel often with three-dimensional diffuseness, although a case of vitreous amyloidosis was previously reported highlighting two-dimensional expansion of opacity localized exclusively to the posterior hyaloid membrane (PHM) [4]. We herein report two cases with idiopathic unilateral diffuse opacification of only the PHM but not the vitreous gel or fluid, together with histological investigations into its possible pathogenesis of gliotic modification to the PHM completely separated from the retina.

## Case presentation

### Patient 1

A 75-year-old female presented with blurred vision of both her eyes gradually worsening for 12 months. The patient had no remarkable personal or family medical history except for Basedow disease in her twenties.

**Fig. 1:**
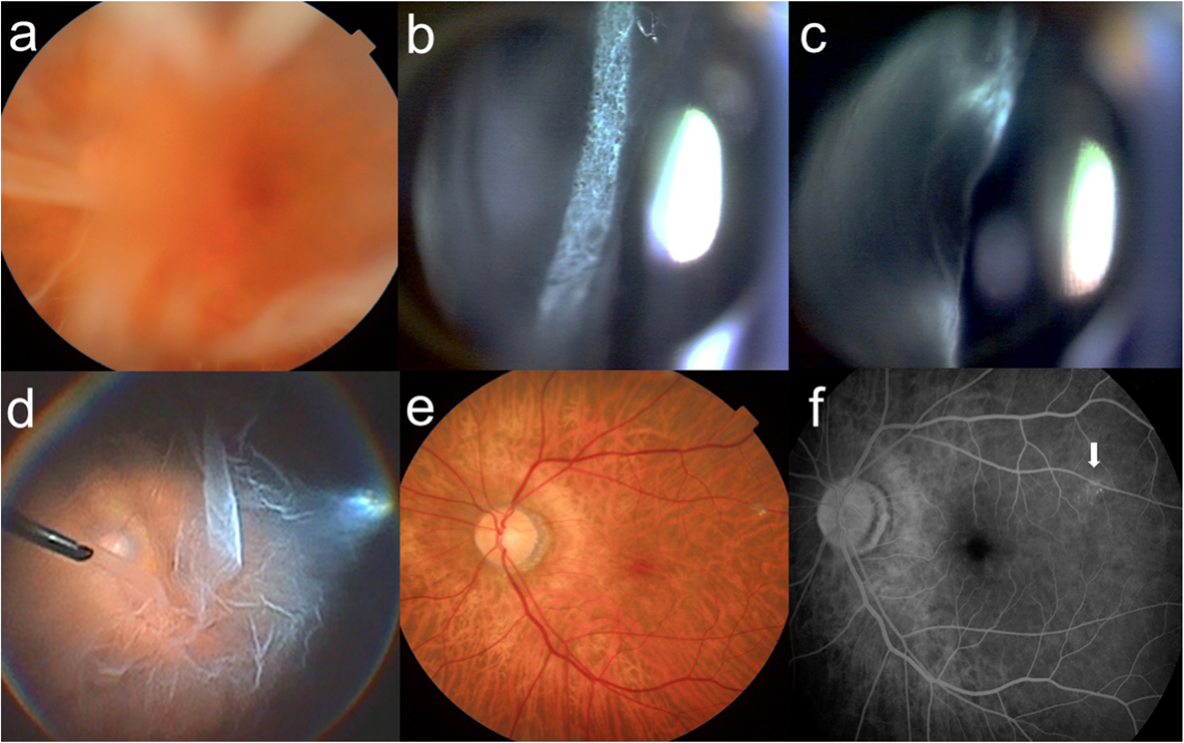
Images of the left eye in a 75-year-old female with vitreous opacity localized exclusively to the posterior hyaloid membrane (PHM) (Case 1). **a** Fundus photograph showing hazy media due to the opaque PHM. **b, c** Slit-lamp photographs showing the PHM opacity resembling a ground glass sheet (**b**), despite clear media with no cellular infiltrates in the vitreous gel or fluid (**c**). **d** Intraoperative image showing the diffusely opaque PHM completely separated from the retina. **e** Postoperative photograph at 12 months showing the normal-appearing retina via recovered fundus transparency. **f** Fluorescein angiography on the late phase showing slight vascular staining with leakage only at the superotemporal arteriole to the macula (arrow)

**Fig. 2:**
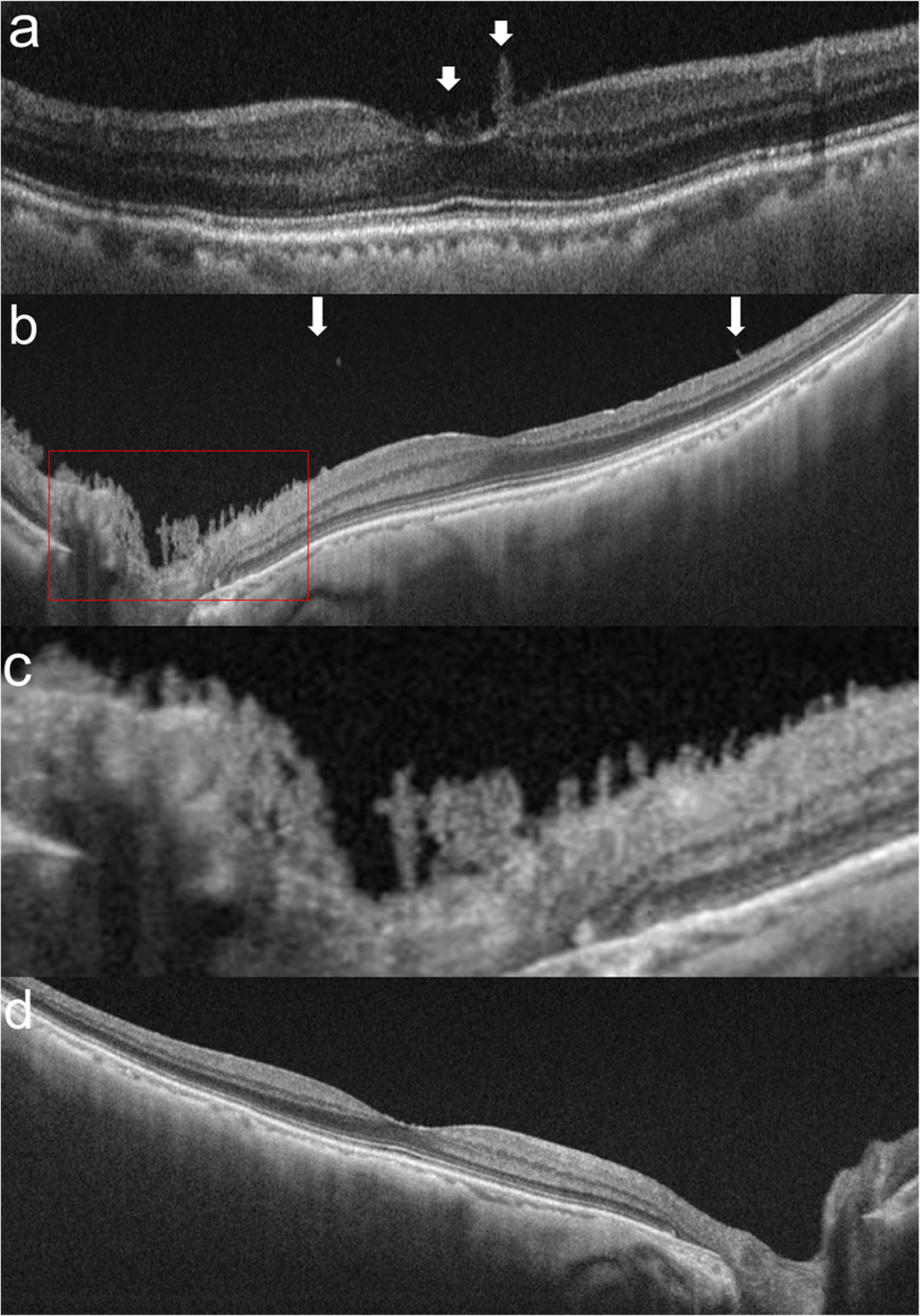
Images of optical coherence tomography (OCT) in the left eye (**a**-**c**) and the right eye (**d**) in Case 1. **a** Preoperative OCT with a 6-mm horizontal scan through the fovea showing hyperreflective icicle-like anterior protrusions from the fovea (arrows). **b** Postoperative OCT at 12 months with a 12-mm scan through the fovea showing hyperreflective icicle-like anterior protrusions on the optic disc and its vicinity. The internal limiting membrane was removed between arrows. C is a magnified view of the red square in (**b**). **d** OCT with 12-mm scan through the fovea and optic disc depicted no abnormalities such as hyperreflective icicle-like structures

Her decimal best-corrected visual acuity (BCVA) was 0.07 OD and 0.2 OS with mild hyperopia. The cornea and anterior chamber were clear OU. Funduscopic examination revealed no obvious abnormal findings via impaired visibility due to severe cataract OU. The patient received cataract surgery with no complication OU, and her BCVA improved to 0.9 OD and 0.6 OS with her complaint of persistent blurred vision OS. Three months postoperatively, the BCVA was almost unchanged, leading to thorough workup to find the cause of persistent blurred vision OS. Funduscopic examination showed no abnormal retinal findings with clear vitreous media OD (Supplemental Fig. 1). Her left fundus was hazy, however, due to diffuse opacity localized exclusively to the PHM resembling a ground glass sheet (Fig. 1a, b) despite clear media with no cellular infiltrates in the vitreous gel or fluid (Fig. 1c). The retina appeared funduscopically normal through the diffusely opaque PHM. Optical coherence tomography (OCT) with a 6-mm horizontal scan through the fovea revealed no abnormal findings OU except hyperreflective icicle-like structures anteriorly protruded from the surface of the fovea OS (Fig. 2a).

She received pars plana vitrectomy with the removal of the diffusely opaque PHM and the internal limiting membrane around the macula. Intraoperatively, the PHM was already completely separated to the periphery from the normal-appearing retina, despite no opacity of the vitreous gel or fluid (Fig. 1d). After the removal of the opaque PHM with a vitreous cutter, triamcinolone acetonide was applied to the retina. Its particles showed some affinity to residual cortex fibrils attached on the internal limiting membrane, which was peeled off with forceps. Use of brilliant blue G confirmed the completion of the procedure because of no staining over the corresponding area. Postoperatively, her BCVA improved to 1.0 OS due to recovered fundus transparency (Fig. 1e). Fluorescein angiography showed no abnormal findings OU except slight vascular staining with leakage only at the superotemporal arteriole to the macula OS (Fig. 1f). Goldmann perimetry showed no abnormal findings OU, and dark-adapted 20 J single-flash electroretinography revealed normal amplitude of a- and b-waves OU (Supplemental Fig. 1). Fourteen months after vitrectomy, her BCVA remained intact with no new retinal or vitreous abnormalities OU. On a 12-mm OCT scan through the fovea, hyperreflective icicle-like anterior protrusions were abundantly observed on the optic disc and its vicinity OS (Fig. 2b,c), whereas no such abnormalities were present at the posterior retina OD (Fig. 2d).

## Patient 2

An 84-year-old female suffered from 12-month-long slowly progressive blurred vision of her left eye. The patient had no significant personal or family medical history.

**Fig. 3:**
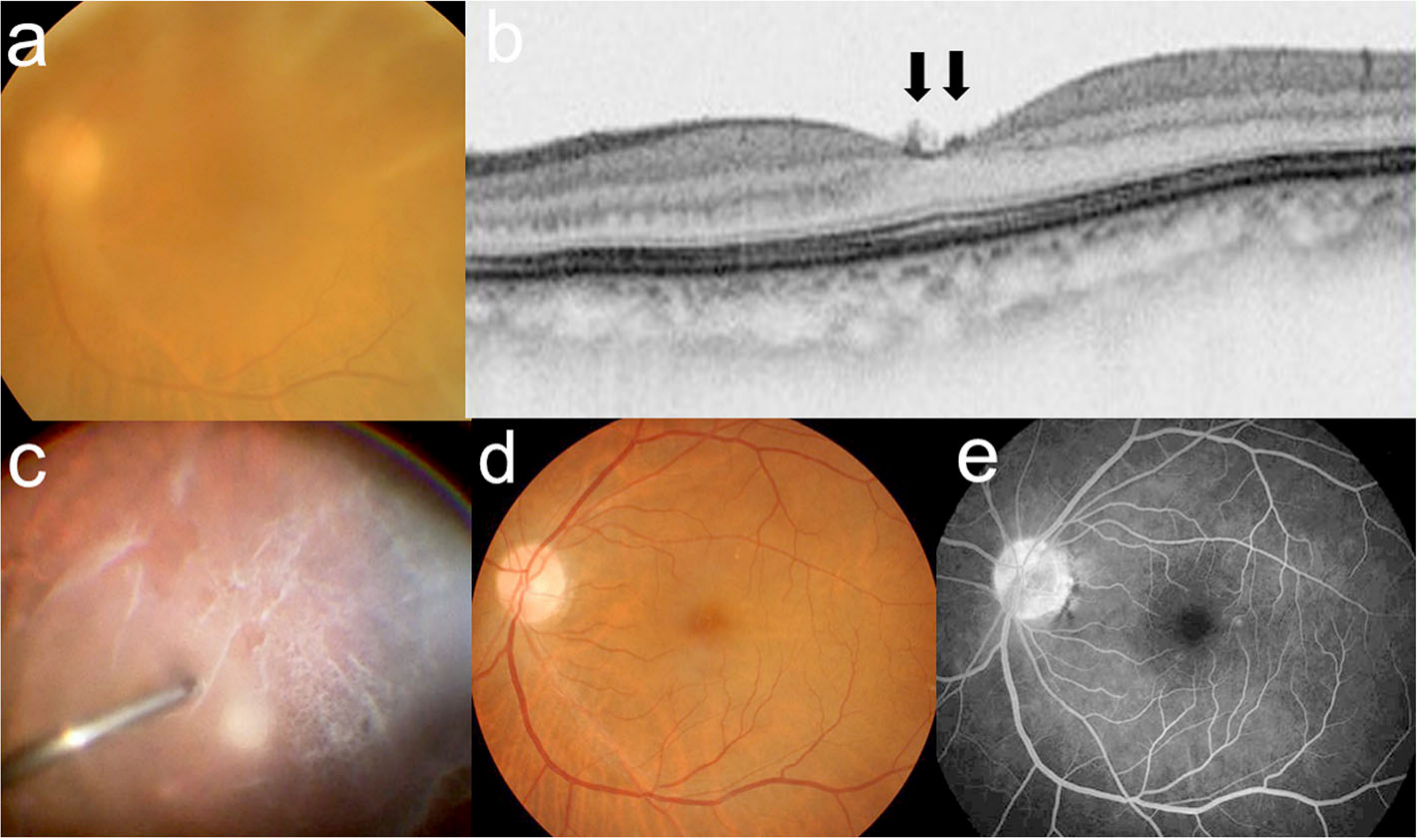
Images of the left eye in an 84-year-old female with vitreous opacity localized exclusively to the PHM (Case 2). **a, b** Preoperative images. Fundus photograph showing hazy media due to the opaque PHM (**a**). Optical coherence tomography showing hyperreflective icicle-like anterior protrusions at the fovea (**b**, arrows). **c** Intraoperative image showing the diffusely opaque PHM completely separated from the retina. **d** Postoperative photograph at 16 months showing the normal-appearing retina via recovered fundus transparency. **e** Late-phase fluorescein angiography showing faint dye leakage from the retinal capillaries as well as the staining of the optic disc

Her decimal BCVA was 0.9 OD and 0.4 OS with moderate hyperopia. The right eye was normal except incipient cataract (Supplemental Fig. 2). In the left eye with incipient cataract as well, there were no other apparent abnormalities in the cornea, anterior chamber, or retina. Her left fundus was hazy due to ground-glass-sheet opacity almost all over the separated PHM, although the vitreous gel and fluid were clear (Fig. 3a). OCT revealed no abnormal retinal findings at the macula OU except hyperreflective icicle-like anterior protrusions on the surface of the fovea OS (Fig. 3b, Supplemental Fig. 2).

She received combined cataract surgery and vitrectomy with the removal of the diffusely opaque PHM OS. Intraoperatively, the PHM was already completely separated to the periphery from the normal-appearing retina, despite no opacity of the vitreous gel or fluid (Fig. 3c). At the surgeon’s discretion, the internal limiting membrane was not peeled off, given that it was sparsely accompanied by residual cortex fibrils stained with triamcinolone acetonide. Postoperatively, her BCVA improved to 1.0 OS due to recovered fundus transparency (Fig. 3d). Dark-adapted 20 J single-flash electroretinography and Humphrey perimetry showed no abnormal findings OU (Supplemental Fig. 2). On fluorescein angiography, however, faint dye leakages from the retinal capillaries were diffusely seen from the posterior retina to the midperiphery, as well as the mild staining of the optic disc OS (Fig. 3e). Sixteen months after vitrectomy, there were no new retinal or vitreous abnormalities OU. The icicle-like hyperreflectivity on the surface of the fovea OS, which was preoperatively detected with OCT (Fig. 3b), remained fairly stable during the follow-up (until her final visit at 16 months postoperatively).

### Histopathology

Cell block preparations of the vitreous taken at vitrectomy were performed for Case 2 according to the previous reports [5, 6]. Briefly, 50 to 100 mL of the diluted vitreous was obtained and transferred to our laboratory at room temperature within 20 min after collection. The diluted vitreous was centrifuged at 2,500 rpm for 10 min. After centrifugation, the supernatants were carefully removed. Cellular pellets were mixed and fixed with 10 % paraformaldehyde overnight at room temperature. The fixed pellets following centrifugation at 3,000 rpm for 3 min were then embedded in paraffin. Five-micrometer unstained sections were made and submitted for staining for hematoxylin and eosin (HE), direct fast scarlet (DFS), and Congo red. Moreover, immunocytochemistry was conducted with an antibody against glial fibrillary acidic protein (GFAP) (1:50; DAKO). Immunoreaction was visualized using 3,3’-diaminobenzidine, and cells were observed using a Biorevo BZ-9000 microscope (Keyence, Osaka, Japan).

**Fig. 4:**
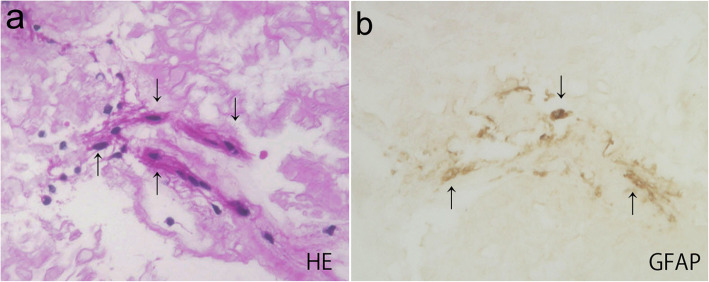
Cell block preparations of the vitreous taken at vitrectomy in Case 2. **a** HE (hematoxylin and eosin) staining showing cluster of mononuclear spindle-shaped cells (arrows). **b** Immunocytochemistry showing cellular components positive for GFAP (glial fibrillary acidic protein) (arrows)

HE staining revealed collections of mononuclear spindle-shaped cells (Fig. 4a), whereas atypical lymphocytes were not seen. The results of DFS staining as well as Congo red staining were both negative for amyloidosis. In contrast, cytoplasmic immunoreactivity for GFAP was clearly noted in the cellular components (Fig. 4b).

## Discussion and conclusions

We encountered two similar cases with the completely separated and diffusely opaque PHM in Japanese females with idiopathic and unilateral onset. The opacification was characterized by ground-glass-sheet appearance with unique localization restricted to the PHM, but not to the vitreous gel or fluid. Immunocytochemistry for GFAP in Case 2 indicated a cluster of activated glial cells, suggestive of gliotic modification to the PHM.

Differential diagnoses should be made in our cases including the following diseases marked by vitreous opacity. Vitreous amyloidosis usually exhibits bilateral glass wool-like opacity deposited disproportionally in places throughout the vitreous gel, on top of wax paper-like opacity attached firmly to the retinal surface [2]. The ground-glass-sheet appearance covering uniformly to the entire PHM separated from the retina appears to be largely different from both of these findings in vitreous amyloidosis. In the literature, there is a rare case report on unilateral vitreous amyloidosis with “lacy-appearing debris adherent to the PHM” which still had apparently disproportional distribution on the PHM surface [4]. The lack of uniformity in vitreous opacity would thus contrast distinctly with our cases. Most importantly, amyloidosis was denied on the basis of negative staining results with both DFS and Congo red. Vitreoretinal lymphoma frequently demonstrates a wide variety of vitreous haze typically known as aurora borealis and a string of pearls, both of which basically result from cellular infiltrates aligned along the vitreous fibrils, on top of orange-yellow subretinal lesions [3, 7]. These findings would again be distinctly different from the ground-glass-sheet opacity showing much more diffuseness and uniformity strictly confined to the PHM surface. Based on the combination of several other negative findings, such as intravitreal atypical lymphocytes on histology, ocular relapse often with systemic involvements [8], and abnormal deposits at multiple retinal layers on OCT [9, 10], vitreoretinal lymphoma was excluded from the differential diagnosis. We could not detect any inflammatory cells in the anterior chamber or the vitreous cavity during follow-up, thus denying the presence of uveitis in our cases.

In the present study, immunohistochemistry on vitreous cell block preparations demonstrated a cluster of GFAP-positive cells, suggesting the migration and proliferation of activated glial cells. The major cellular components of epiretinal proliferative tissues include glial cells that induce fibrosis and/or gliosis, the former of which are mediated by myofibroblastic transdifferentiation from glial cells, also known as glial-mesenchymal transition (GMT) seen in idiopathic epiretinal membrane [11] and proliferative diabetic retinopathy [12]. GMT is characterized by focal contractile forces exerted by transdifferentiated myofibroblasts with massive collagen production (i.e., fibrosis), generating funduscopically visible wrinkles and folds on the membrane, which would however be apart from the ground-glass-sheet appearance free of such focal contraction in our cases. In contrast, gliosis is another known scarring mechanism basically devoid of collagen production and tissue contraction, whereby nondifferentiated glial cells proliferate with enhanced formation of GFAP-positive intermediate filaments exerting no contractile force. Glial cells undergoing GMT tend to lose glial property with GFAP downregulation, while acquiring myofibroblastic features such as contractile smooth muscle actin overproduction [11]. The PHM resembling a ground glass sheet would reasonably stem from gliosis due to lack of focality as well as contractility, in combination with the presence of a cluster of cells strongly immunopositive for GFAP. Although the cytology-proven features (gliosis but not amyloidosis or lymphoma) resulted from a single-case assessment, it might actually be supportive for the presumed pathogenesis of the other case, given the peculiarity and commonality in clinical appearance of the present two cases.

The etiology of the present cases remains largely unknown because no explainable backgrounds, whether systemic or ophthalmic, could be identified so far. The two patients inflicted no occupational exposure to toxic metals or organic solvents, and shared neither genetic relationship nor any particular living environment. It would be interesting to note, however, that there were two common ocular findings in the affected eyes of both patients: subclinical vascular leakage barely detected with fluorescein angiography and icicle-like anterior protrusions clearly presented with OCT. Although these two abnormalities may have coexisted coincidentally, a possible mechanistic link is likely to be inflammation-driven migration of some undetermined retinal cells or resultant deposition of cellular debris. From lack of further evidence clinically collected, it is too speculative at present to state that such minimal vasculitis causes glial cell migration out of the retina onto the entire PHM, given that the affected eyes exhibited no signs of active or convalescent uveitis. We did not check any inflammatory cytokines such as chemotactic factors. Nevertheless, the two retinal findings associated with gliotic opaque PHM separation would definitely be worth considerable attention in terms of mechanistic insight. These limitations in the present study warrant future and further investigations into other similar cases.

In conclusion, to the best of our knowledge, this is the first to report such peculiar cases of ground-glass-sheet opacity confined diffusely to the PHM surface completely separated from the retina. Immunopositivity for GFAP suggested, at least in one case, gliotic modification to the pathogenesis of this undocumented vision-threatening disorder, leading us to propose its nomenclature “gliotic opaque PHM separation” as a new clinical entity.

## Supplementary Information

**Supplemental Figure S1** Ophthalmological findings in Case 1. Top left Fundus photograph showing normal appearance OD. Top right Late-phase fluorescein angiography showing no abnormal findings OD. Bottom left Goldmann perimetry showing normal appearance except for slight enlargement of a blind spot OS. Bottom right Dark-adapted 20 J single-flash electroretinography showing normal amplitudes OU. 

**Supplemental Figure S2.** Ophthalmological findings in Case 2. Top left Fundus photograph showing normal appearance OD. Top right Late-phase fluorescein angiography showing no abnormal findings OD. Middle OCT showing no abnormal findings at the macular region OD. Bottom left Humphry perimetry showing normal appearance OS. Bottom right Dark-adapted 20 J single-flash electroretinography showing normal amplitudes OU.

## Data Availability

All data generated or analyzed during this study are included in this published article.

## Abbreviations

BCVA: Best-corrected visual acuity
DFS: Direct fast scarlet
GMT: Glial-mesenchymal transition
HE: Hematoxylin and eosin
GFAP: Glial fibrillary acidic protein
OCT: Optical coherence tomography
PHM: Posterior hyaloid membrane
