# Supplementary figures and images for: Gliotic opaque posterior hyaloid membrane separation: report of two cases
Source: BMC Ophthalmol. 2021 Aug 23;21:308. doi: 10.1186/s12886-021-02072-5 (PMC8381526; doi:10.1186/s12886-021-02072-5)

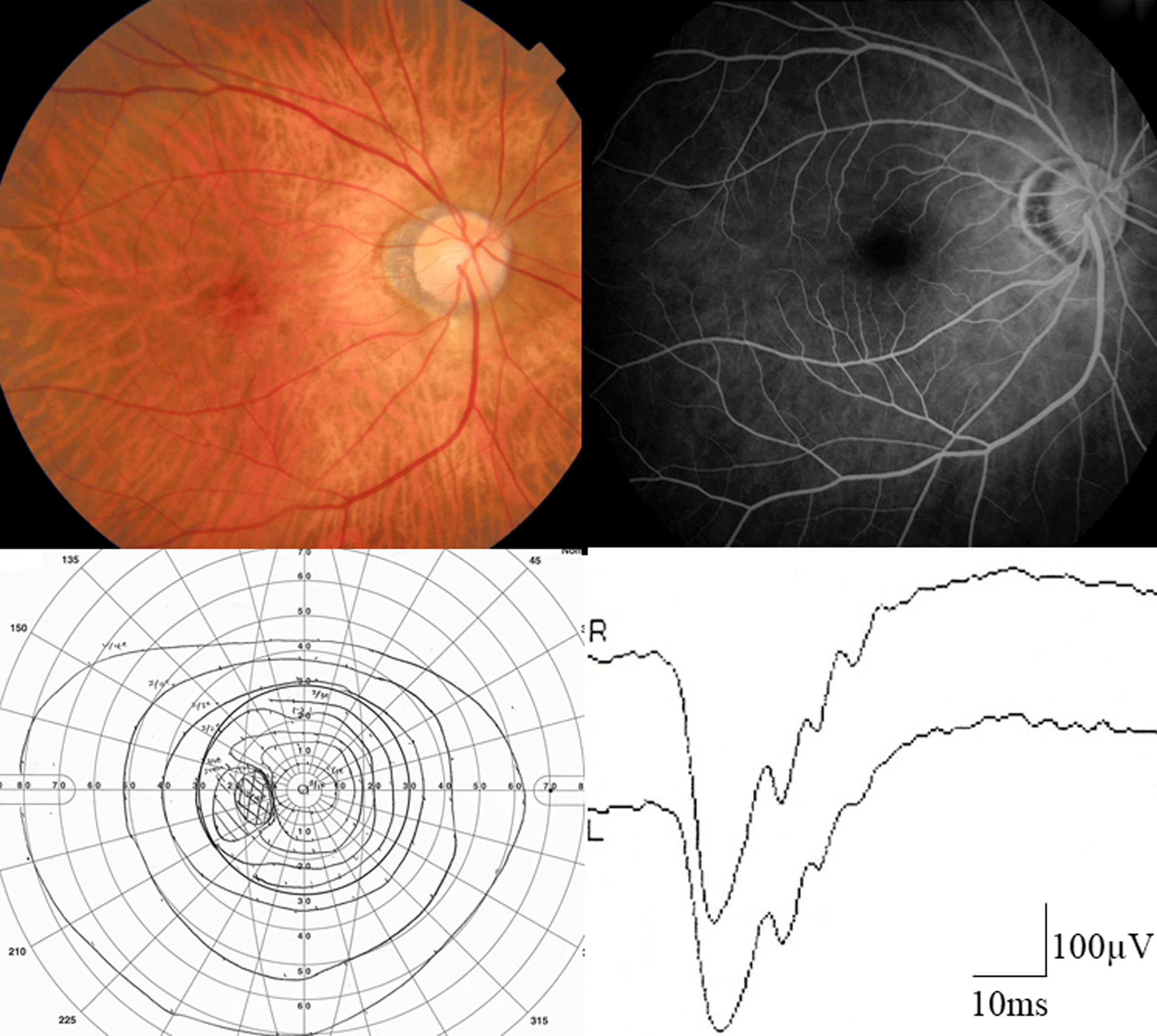

Supplement: Supplementary file 1 — Supplemental Figure S1 Ophthalmological findings in Case 1. Top left Fundus photograph showing normal appearance OD. Top right Late-phase fluorescein angiography showing no abnormal findings OD. Bottom left Goldmann perimetry showing normal appearance except for slight enlargement of a blind spot OS. Bottom right Dark-adapted 20 J single-flash electroretinography showing normal amplitudes OU. [file 12886_2021_2072_MOESM1_ESM.tif]

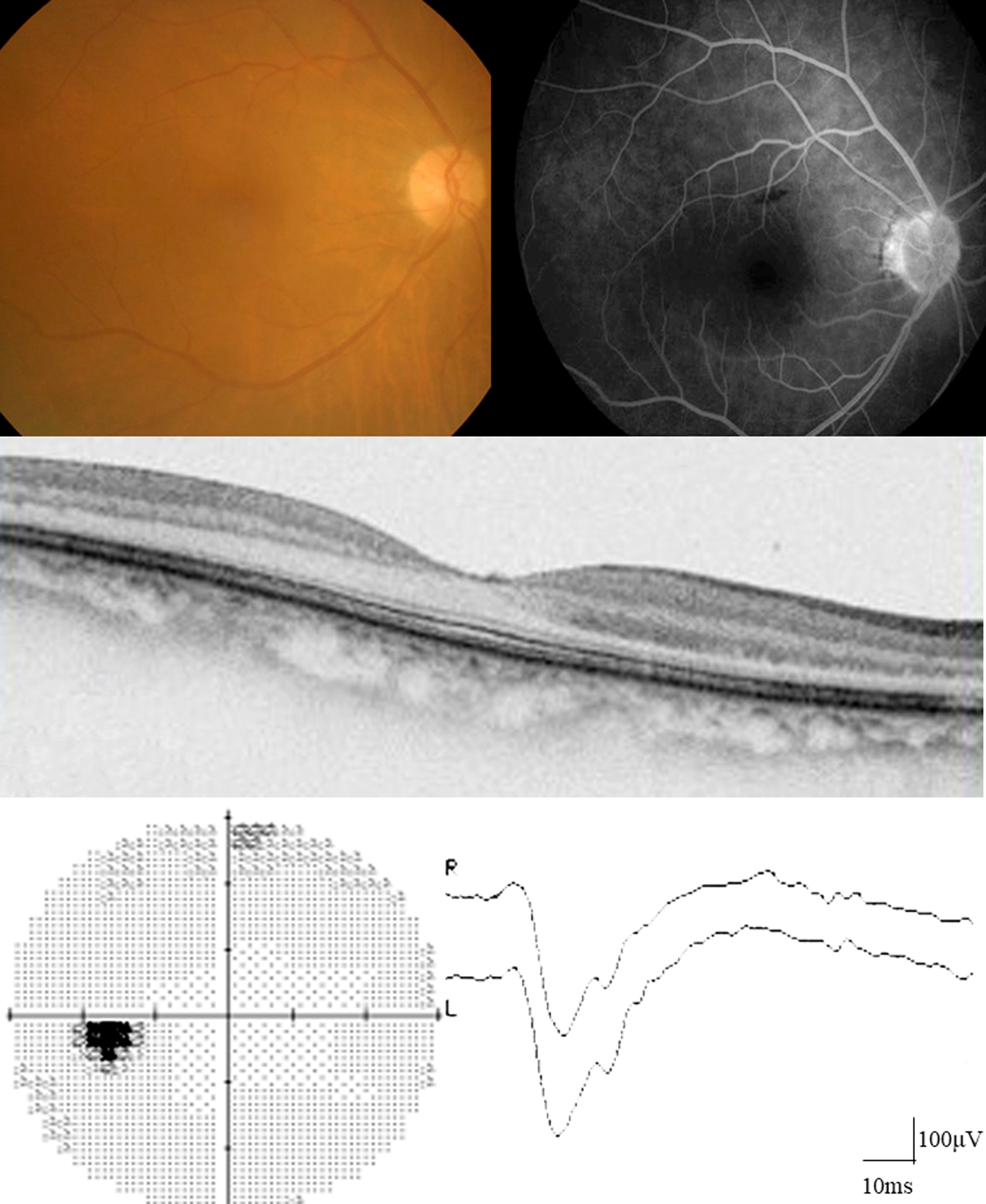

Supplement: Supplementary file 2 — Supplemental Figure S2. Ophthalmological findings in Case 2. Top left Fundus photograph showing normal appearance OD. Top right Late-phase fluorescein angiography showing no abnormal findings OD. Middle OCT showing no abnormal findings at the macular region OD. Bottom left Humphry perimetry showing normal appearance OS. Bottom right Dark-adapted 20 J single-flash electroretinography showing normal amplitudes OU. [file 12886_2021_2072_MOESM2_ESM.tif]
